# Total Design in the Design and Development Process of a Remotely Operated Vehicle (ROV) with Particular Consideration of Sensorization

**DOI:** 10.3390/s22093284

**Published:** 2022-04-25

**Authors:** Teresa Ramos, Antonio Córdoba, Amalia Luque, Ana de las Heras

**Affiliations:** Department of Design Engineering, Higher Polytechnic School, University of Seville, Virgen de Africa, 7, 41011 Sevilla, Spain; tramos@us.es (T.R.); acordoba1@us.es (A.C.); amalialuque@us.es (A.L.)

**Keywords:** remotely operated vehicle, Pugh’s Total Design model, product development process, industrial design engineering

## Abstract

This paper provides a methodological proposal for the design and development process of a remotely operated vehicle (ROV). The design core and product design specifications (PDS) of Pugh’s Total Design model are considered, with a focus on the early stages of the product design and development process. A modularization of the functional groups of an ROV is proposed, focusing attention on the sensor system. The main concepts regarding ROVs are presented, Pugh’s Total Design model is explained, justifying the application interest in technological projects, a methodological proposal adapted to ROV projects is provided, based on Pugh’s Total Design model, with special interest in the early stages of the new product development process (NPD), the suitability of applying our own model of industrial design engineering in an ROV system is analyzed, and the contribution of this study is evaluated, proposing future work and lines of research.

## 1. Introduction

The marine environment is known as one of the most unexplored scenarios by human beings, historically demonstrating great interest in exploration and research according to the discoveries obtained as a result of underwater expeditions [1]. The evolution of oceans and seas in the face of alterations in the natural order such as climate change and the melting of the polar ice caps has reinforced the importance of developing new technologies that enable the exhaustive analysis of water, relief and marine biodiversity (species macroscopic and microscopic) [2].

Remotely operated vehicles (ROVs) are a technological solution that allows the development of a wide variety of underwater tasks such as the collection of audiovisual material, data for the parameterization of the environment through sensors, sample collection, infrastructure monitoring and inspection [3], environmental prospecting [4], biodiversity studies [5], etc. For this reason, there are various fields of application that make use of these vehicles and invest efforts in the development of ROV systems to carry out specific operations, improve technologies, etc. [4]. Since the 1950s, the technology associated with the ROV sector has been developed especially by the military and oil and gas industries, demonstrating the potential of these vehicles that Macreadie et al. [6] referred to as “eyes in the sea”.

### 1.1. Background

**Figure 1:**
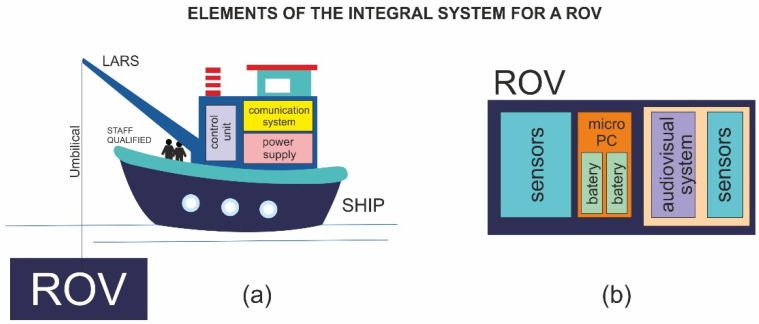
(**a**) Graphic representation of an ROV system. (**b**) Main subsystems of an ROV. Based on [12,13].

In general, a remotely operated vehicle (ROV) is called an unmanned underwater vehicle (UUV), and is connected to a surface vessel, from which it is controlled remotely by the human operator, allowing the development of specific tasks. In turn, the ROV forms part of an integral system, called the ROV system (Figure 1), consisting of [7]:

- Vessel: this is mainly where the central control cabin is located, from which the pilot controls the ROV, the vessel power and the launch and recovery system (LARS) for large ROVs.
- Umbilical: this is a connector that connects the vehicle with the surface, allowing the supply of energy and the transmission of information. In ROVs of considerable size, the tether management system (TMS) must be considered, which facilitates the launch and recovery of the vehicle and provides protection to the vehicle during its launch and recovery.
- ROV: this is the vehicle in charge of carrying out tasks in the environment. This will have technical characteristics such as depth of reach, speed, battery autonomy, weight, payload, according to the application to which it is intended.

In general, UUVs are classified based on the connection system with the surface, integrating two categories, ROVs and autonomous underwater vehicles (AUVs). ROVs are controlled manually by a human being remotely through the umbilical cable, which can limit the range of the ROV, but they have the advantage of communicating in real time with the central control cabin thanks to the umbilical connection; AUVs are not manually controlled, and rather their trajectory is programmed and they operate autonomously [8]. In turn, ROVs can be classified based on the vehicle’s energy source (surface-powered, vehicle powered and hybrid system), the degree of autonomy (fully autonomous, semi-autonomous, tele-operation and remote control), communications link (hard-wire, acoustic, optical and radio frequency communications) [9], their dimensions (intervention-class ROVs and work-class ROVs) and the depth at which they can work [10].

In general terms, an ROV is equipped with a multitude of elements for monitoring, such as camera systems, sonars, sensors, positioning systems, and other specific functional groups according to the application of the vehicle (Figure 1). As for the functional groups that an ROV includes, the following can be considered in general terms: frame system, buoyancy system, propulsion system, sensor system, lighting system, audiovisual system, geolocation system, power and connection system [11,12].

There are recent studies on the applications of ROVs that demonstrate their usefulness for research purposes, as well as for other industries within the maritime sector as diverse as deep sea mining, aquaculture and the marine renewable energy industry [14,15]. In relation to environmental and oceanographic research projects, ROVs allow the collection of a variety of data on the seabed and biodiversity in a precise manner, for purposes such as the determination of marine protected areas and the definition of conservation aspects [16,17]. In marine archaeology, they are used for the exploration of underwater pools of toxic brine and the observation of underwater structures [18]. In the industrial sector, ROVs allow the performance of underwater interventions, repair and maintenance operations in structures belonging to the oil and gas industries, marine sciences, aquaculture, naval defense, marine renewable energies, networks and wiring, among others [19,20].

The rise of this type of vehicle and the variety of industrial and research applications available demonstrate the importance of proposing a systematic methodological framework specific for the discipline of industrial design engineering that helps professionals to project the entire product development process (PDP) of ROVs.

### 1.2. Literature Review

The versatility of ROVs in terms of applications and effectiveness has forced companies to seek not only functional improvements in terms of autonomy and precision, but also to focus on reducing costs to obtain more affordable underwater vehicles. Capocci, Dooly and Toal [21] developed an inspection ROV for the offshore renewable energy sector with the aim of reducing operational and maintenance costs. Its value proposition lies in a “hybrid” design between a lighter inspection-class ROV and a larger work-class ROV that needs a LARS system. The initial design process is based on integrating a system of sensors and advanced control structures from the selection of specific components seeking maximum propulsion capacity. The applied design methodology consists of an analysis of the different functional groups, which are gradually defined by adapting to the final set: frame and buoyancy, thrusters, power system, communication and equipment, enclosure system, sensor system and control system.

In turn, the rise of underwater vehicles such as ROVs and AUVs has greatly boosted the technological effort in sensing, for example in looking for new sensors for in situ marine detection and the collection of information and water parameters in regions that are not very inaccessible. The latest advances in the field of sensorics applied to underwater robotics include fiber optic-based sensors that provide temperature and pressure (depth) measurements with significant advantages over other existing sensor technologies [22]. Another important branch is analytical sensorics based on laser applications, such as laser-induced breakdown spectroscopy (LIBS) and Raman, which allow solid sample on-site analysis [23,24].

As for ROV sensors, fiber optic depth sensors that combine pressure and temperature measurement, providing stability values and optimal performance, are being developed [22]. Other applications have been found such as the use of acoustic sensors for the characterization of the noise emitted by underwater vehicles with the aim of maximizing the quality of the acoustic data recorded by the ROV [25]. Sensors are introduced to increase the security of submarine communication cables (ROVS and AUVs), useful in detecting threats such as divers, anchors, fishing nets, submarines and other vehicles [26]. It is of interest to note the introduction of electromagnetic sensors capable of calculating the range and speed of the vehicle accurately to ensure the safe docking of AUVs [27].

On the other hand, the use of the Internet of Things is being introduced for the rapid monitoring of water quality parameters based on information from sensors that measure pH, turbidity (NTU), and substances that are dissolved in the water [28].

There are other research areas focused on the development of kinematic and dynamic models in the underwater environment, which can be individualized according to the configuration, dimensions and buoyancy of each vehicle. There is great interest in innovation in propulsion systems in response to the need to obtain a flexible and reconfigurable ROV in its movement through the marine environment. The development of these propulsion systems offers the ROV degrees of freedom (DOF), reaching six DOF in linear and non-linear models [29,30]. To define the ROV’s trajectory, optimization algorithms are being implemented using 3D dynamic models that improve the ROV’s exploration capacity [31]. From this area arise projects for the design of the control system focused on detecting and minimizing failures of the propulsion system and ensuring the stability of the ROV in the underwater environment through the fault-tolerant control (FTC) method that considers the disturbances of the model induced by failures in propellants [32]. There is also research on the design and development of event-driven fault detection filters for discrete time in multi-agent linear systems that collaborate in the detection of faults in the system [33].

The focus of current research is centered on the development of new sensory devices that optimize the tasks of the ROV, as well as the rest of the systems. The development of the investigations begins in the stages of preliminary design and detailed design of the physical domain or of the components, with a great interest in the simulation and optimization of said devices, which serves as motivation for our research work. There is a lack of study of the early stages of the PDP of ROV such as the analysis of user and market needs, definition of the product design specifications (PDS) and conceptual design, thus assuming a research gap that this work intends to address.

### 1.3. Objectives

This research provides a methodological proposal for the design and development process of ROVs considering the design core and the PDS of Pugh’s Total Design model [34], focusing attention on the early stages of the PDP. A modularization of the sensor system is proposed, which could be extrapolated in future works to the rest of the functional groups of an ROV. As particular objectives, the following points are raised:

- Examining the state of the art of the design and development of ROVs.
- Studying methodologies of engineering design
- The proposal and application of Pugh’s Total Design model to a ROV design and development project.

This study is structured including the following sections: Section 1, where the main concepts of ROVs are explained according to the bibliographical analysis and the proposed objectives are specified. In Section 2, Pugh’s Total Design Model is presented, justifying the interest in its application in technological projects and the bibliographic review methodology. In Section 3, a methodological proposal adapted to ROV projects is presented, based on Pugh’s Total Design model, with special interest in the early stages of the PDP, and a modularization of the ROV sensor group is proposed. Finally, in Section 4, the suitability of applying our own model of industrial design engineering in an ROV is evaluated, proposing future works and lines of research.

## 2. Methods and Materials

A design methodology is understood as those procedures, techniques and tools that a designer engineer can use in the design process and general development of a product [35]. These methodologies propose a structure that includes all the phases of the PDP, from the moment the problem is established and information is collected until the obtaining and definition of a successful solution.

Nigel Cross [36] established a classification based on descriptive and prescriptive models. Prescriptive design models focus on providing guidelines, rules and procedures, as well as establishing guidelines to develop each of the stages of the design process in order to help design engineers [37]. These types of models are based on systematic processes, beginning with the analysis of the problem and avoiding preconceptions. Examples of prescriptive models are the Pahl and Beitz model [38], Pugh’s Total Design model [34], and Archer’s model [39], among others, which are predominant in engineering. Descriptive design models treat the design process from a cognitive point of view, structuring the problem in a cyclical way starting with solution guesses. Some of the descriptive models include the linear descriptive model and Cross’ model [36], among others, which are predominant in architecture [40]. Different authors have addressed this issue by proposing other classifications, such as descriptive models, prescriptive models and computer-based models [41].

**Figure 2:**
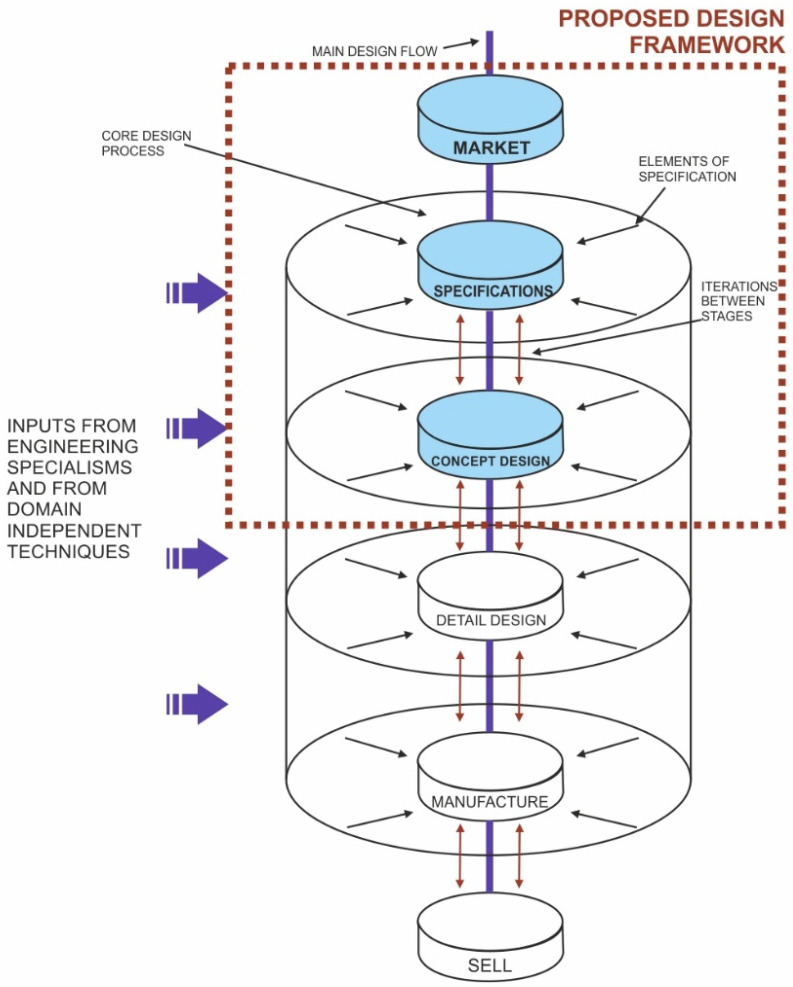
Graphic representation of the Pugh’s Total Design model. Based on [34].

Within the prescriptive models, Pugh’s Total Design is that which performs a more detailed deployment of the PDS in the early stages of the PDP. These PDS are obtained directly from the analysis of market and user needs and are the starting point of the conceptual design phase. The rest of the models, such as the Pahl and Beitz model, define the existence of design specifications but do not offer a pre-established list of PDS, as in the Total Design model (Figure 2). In the field of ROV design, PDS are very important due to the variety of applications and research areas offered by this type of vehicle.

Research on the early stages of the PDP of ROVs starts from an already established physical domain, without previously defining needs, PDS and functions [15,42]. These investigations focus attention on the development of the propulsion system, data and power system [43]. Satria et al. [44] used the Pahl and Beitz model to structure the problem and perform a selection of criteria using Pugh’s Concept Screening Matrix, although they did not delve into the definition of PDS or offer a complete view of the PDP of ROVs.

There is no consensus on how to address the first stages of the PDP in ROVs; it is common to use block diagrams to structure the physical domain of the ROVs, providing information on the components and their future structure [15,45]. In the early stages, it is common to obtain lists of specifications either proposed by the authors themselves or obtained through expert surveys [46,47]. In the trend analysis on the design and development of ROVs, it is specified that one of the techniques is the application of modular design principles [48] and the introduction of bio-inspired design techniques [49]. In the detailed design phase, the use of tools such as computational fluid dynamics (CFD) and finite element analysis (FEA) is more established [47]. This research aims to contribute to the development process of ROVs proposing a systematic methodological framework specific for the discipline of industrial design engineering that helps professionals to project the entire PDP of ROVs.

### 2.1. Total Design

Based on the objectives established in the research, the incorporation and adaptation of Pugh’s Total Design model in ROV design and development projects is proposed as a reference model within the framework of concurrent engineering. This model provides a systematic methodology to achieve the integration of those aspects, not only technological, involved in a product design and development project. Pugh [34] establishes two main elements in the model:

- The design core encompasses six design cores belonging to the six stages integrated in the model and whose graphic representation is made vertically with several links that correspond to each stage (Figure 2). The established stages are: core design 1: market/user needs and demands, core design 2: the product design specification, core design 3: conceptual design, core design 4: detail design (technical design), core design 5: manufacture and core design 6: selling (marketing). These stages help the engineer or design team to treat the PDP systematically, being able to adapt the methodology to the product.
- PDS refer to technical, market, manufacturing, etc., considerations, and their objective is to limit those parameters and main requirements in the product, preparing a list with qualitative or quantitative specifications in the form of limit values or specific values if they are known. Each phase defined in the design core has its own PDS which are represented around the stages and ordered based on their importance for the stage (Figure 2). The design core enveloped by the PDS thus integrates the activities systematically to structure the PDP.

In the first stage, called “market”, an investigation is carried out regarding the product to reach an understanding of the market situation and the needs of the user, as well as the recognition of competing products.

**Figure 3:**
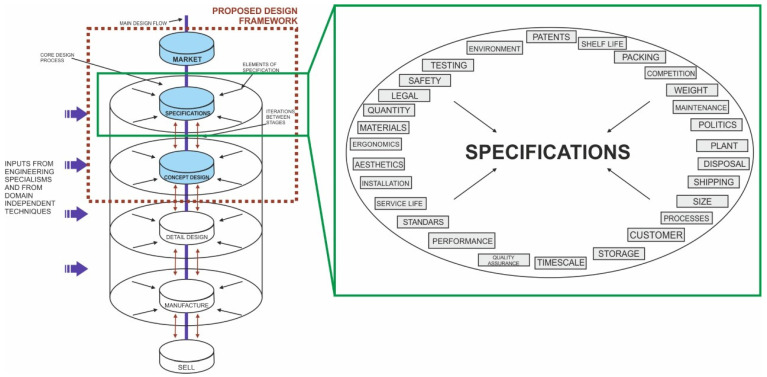
Product design specifications (PDS) of Pugh’s Total Design model. Based on [34].

The next stage, called “specification”, is of vital importance in the process as it directly controls the subsequent design stages, and by extension, the final result. It is a dynamic stage in which it is sought to delimit those parameters and main requirements in the product in the form of a list with qualitative or quantitative specifications that can include limit values or specific values if they are known. The definition of specifications will be worked on in detail, avoiding ambiguity, since these will be the guides of the process and will define the success of the product. For the definition of the PDS, it is useful to have a broad approach encompassing all phases of the product life cycle, a recapitulation of the states of the system and key parameters that it experiences. Figure 3 shows the PDS proposed by Pugh [34] in their Total Design model for an overall product design and development project. The result of this stage would be the list of PDS that will intervene in all the stages of the process.

Once the specifications stage has been established by defining the PDS, we proceed to the conceptual stage of design. This is a synthesis stage in which the generation of ideas and solutions is worked on with respect to the needs defined in the “market” stage and having as input the information defined in the “specification” stage. In the conceptual stage of design, solutions must be devised to comply with the PDS. Integrated activities in this stage include the classification and formulation of the problem and the search and exploration of solutions through creative techniques. Once the design solutions have been obtained, decision-making techniques must be applied to select a design solution and validate it with respect to the reference market, this being the result of said stage.

In the stage defined as “detail design”, the design concept resulting from the previous stage is defined, in relation to technology, materials and processes. As a result of the application of this stage, the layout and the physical or component domain of the product must be defined, as well as a cost analysis and the pertinent analyses based on the product design (stress analysis, impact analysis …). At this stage, it will be useful to describe the component design specifications (CDS) to satisfy the PDS.

For the development of the design core manufacturing stage, design for manufacture techniques must be considered to design the manufacturing process. Regarding the PDS, it is important to apply optimization techniques to obtain a good design, since when optimizing one element of the PDS, others may be altered. The last design core, called selling (marketing), must start from the characteristics of the product and the needs of the users established in the first design core. In this stage, the marketing of the final product is carried out, defining distribution systems, service activities and everything related to marketing and sales activities.

### 2.2. Review Methodology

To carry out the bibliographic study, the guidelines established by Mayer [50] were followed, as well as their revision classification proposal. The review was structured in different stages: definition of the problem (Section 1.3), search for information (Section 2.2), organization of information and analysis of results (Section 3.1). We proposed carrying out a bibliographic review of the state of the art type, collecting the most current advances in a specific topic or field of research. The review firstly focused on the study of the concept of industrial design methodologies applied to ROV design and development projects, and secondly, on the relationship of ROV projects with Pugh’s Total Design model, all with a special interest in the sensors incorporated in these vehicles.

The realization of the state of the art was focused on the analysis of the most relevant publications in the scientific literature according to the proposed approach. The bibliographic search was limited to a time span of five years, from 2016 to the present, evaluating the content, authors and scope of knowledge of the publications. The criteria proposed for the selection and classification of the literature were:

- Time criterion: search and analysis of publications produced between 2016 and 2021.
- Context of the concept: articles focused on ROVs were selected, with special interest in those dealing with sensorics in ROVs.
- Methodological framework criteria: special interest was given to publications in which the PDDP of an ROV is structured following some design model or methodology from the early stages of the process.

To define the bibliographic search terms, a preliminary scoping study was carried out to examine the breadth of the bibliography around the design and development of ROVs. The results of this scoping study helped us to iteratively refine search terms to minimize articles unrelated to the PDP. The final search terms were: (“Remotely operated vehicle” OR “ROV”) AND (“design methodology” OR “Pugh’s Total Design”).

## 3. Results and Discussion

**Figure 4:**
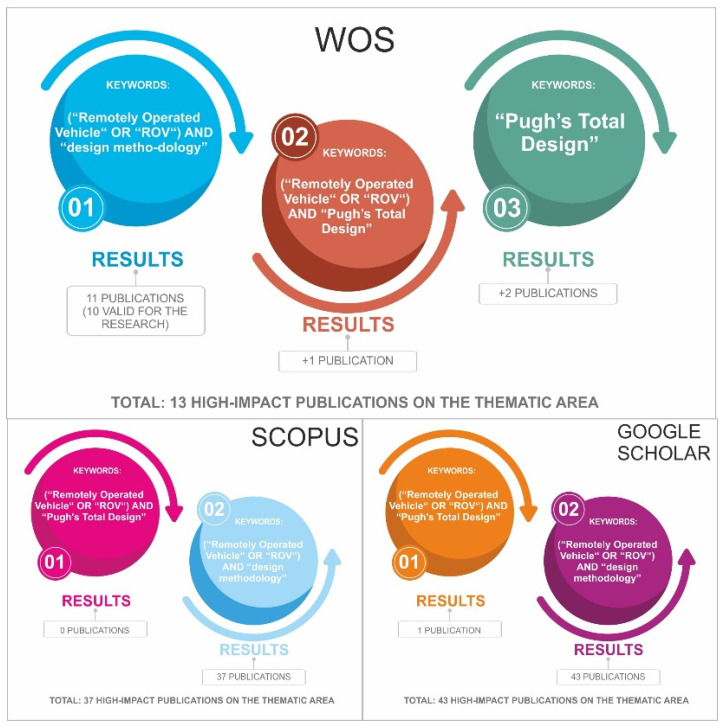
Proposal and results of bibliographic review.

The bibliographic analysis carried out included the search in Web of Science (WoS) focused on the terms (“remotely operated vehicle” OR “ROV”) AND “design methodology” with a total result of 11 references from 2016 to 2021 (Figure 4). This search was expanded by incorporating the proposed design model “Pugh’s Total Design” using as search terms (“remotely operated vehicle” OR “ROV”) AND “Pugh’s Total Design”, finding two more references, which denote a niche of research in the development of ROVs from the field of industrial design engineering. On the other hand, a bibliographical analysis was carried out in Scopus where the search (“remotely operated vehicle” OR “ROV”) AND “Pugh’s Total Design” did not obtain results, and (“remotely operated vehicle” OR “ROV”) AND “design methodology” obtained 37 publications between the years 2016 and 2021. The Google Scholar search for (“remotely operated vehicle” OR “ROV”) + “Pugh’s Total Design” returned one result, and (“Remotely Operated Vehicle” OR “ROV”) AND “design methodology” obtained 43 results.

**Figure 5:**
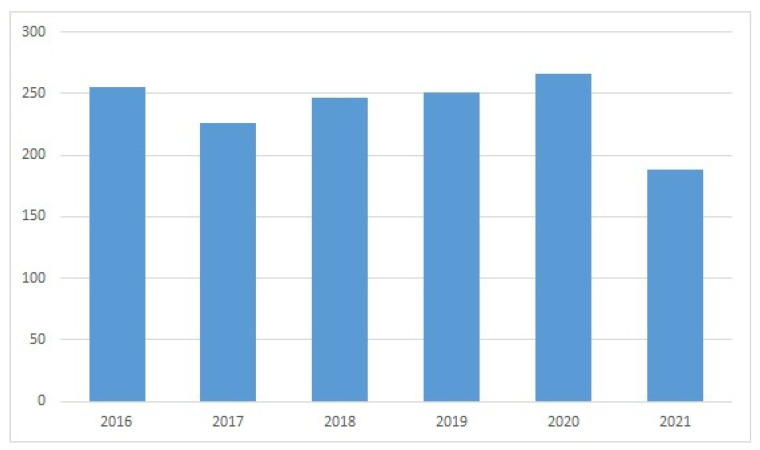
Evolution of publications in WoS for “ROV” (year, number of publications).

**Figure 6:**
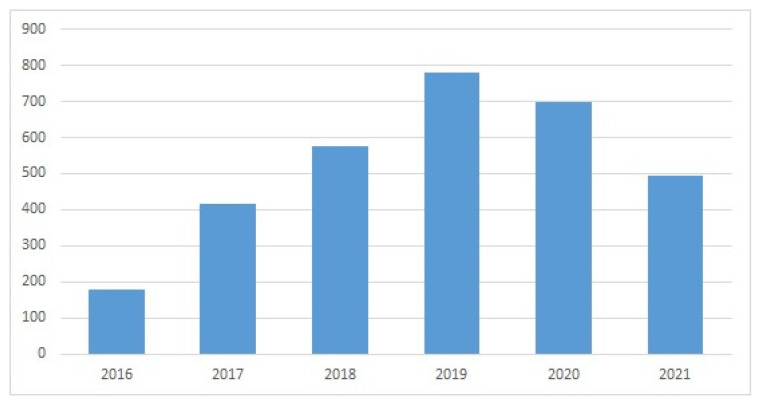
Evolution of publications in WoS for “remotely operated vehicle” AND “design” (year, number of publications).

**Figure 7:**
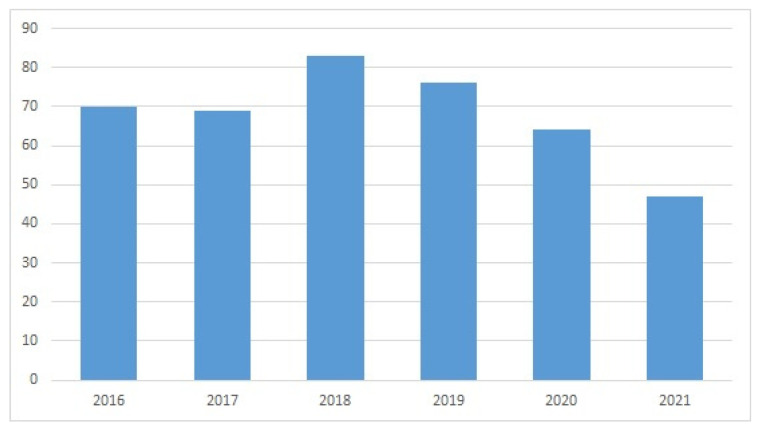
Evolution of publications in WoS for “ROV” AND “design” (year, number of publications).

**Figure 8:**
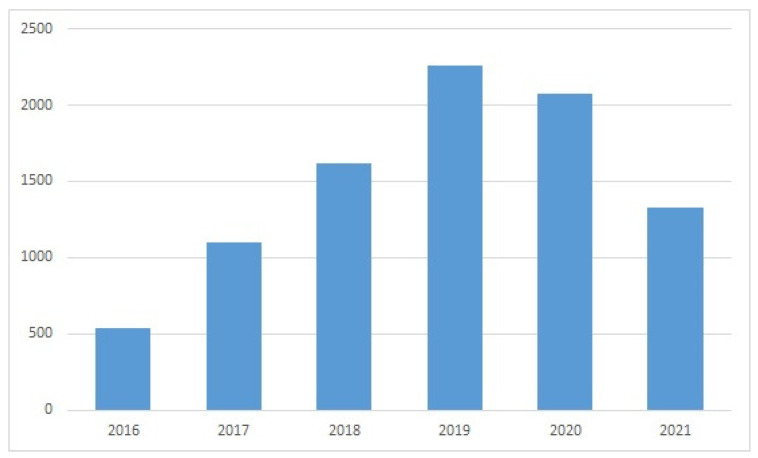
Evolution of publications in WoS for “remotely operated vehicle” OR “ROV” AND “design” (year, number of publications).

The WoS search provided evidence of a sustained interest in subsea devices and ROVs in recent years (Figure 5, Figure 6, Figure 7 and Figure 8). The wide variety of applications of ROVs in the maritime sector has been decisive for the development of implemented technological advances and those that are currently still being studied and tested.

The results obtain evidence of the existence of research in the design and development of ROVs focused on specific requirements of the detailed design stage or on the definition and optimization of specific subsystems of the vehicle. No studies have been found that include and delve into the early stages of the project or the initial phases of the PDP. This situation presents a research opportunity that improves the organization and application of phases such as market research, definition of specifications and conceptual design in ROV design and development projects.

Thanks to this organization, design models for ROVs could be proposed as future work, customized for industrial sectors or specific research areas. Other market segments could also be addressed, developing designs focused on non-expert users, in the recreational or sports field.

The design and development process in engineering requires a holistic approach, with the Total Design model being ideal for synthesizing the main activities together with recurring aspects in engineering projects in the form of PDS. In this case, and based on the results obtained, attention will be focused on the “market”, “specification” and “concept design” stages. The design and development process of ROVs can encompass multiple requirements and subsystems, from structural and mechanical design, electrical and electronic system, sensor system, product lay-out, communication system and supply with the control command [15]. In this proposal, attention will be focused on the ROV sensing system.

### 3.1. Total Design in ROV System Design and Development

Given the broad classification of ROVs depending on the application for which they are intended, the scope of this study will focus on ROVs for research work. Next, a proposal is made based on the Total Design model for the definition of the ROV, focusing on the sensor system.

#### 3.1.1. Design Core: Market

**Figure 9:**
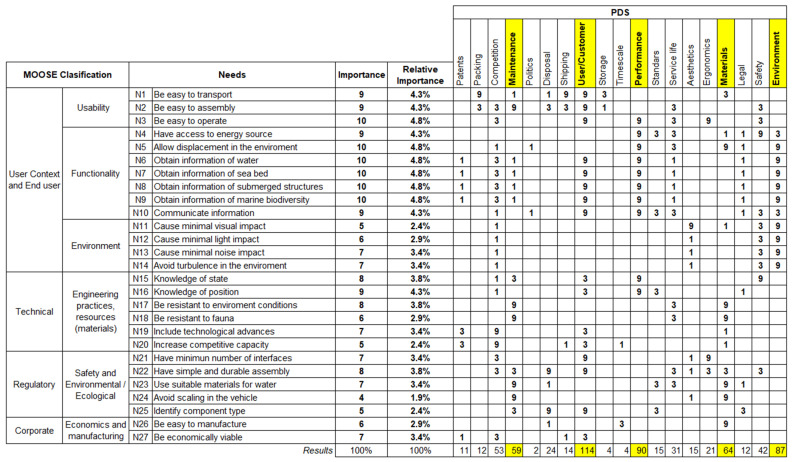
Matrix of relationships between needs and ROV PDS.

To address this stage, we propose carrying out an analysis focused on the needs of users regarding an ROV. To carry out a needs analysis, there are two main approaches, the market approach and the product approach [37]. The ROV, being a highly technological product and not being a consumer product (final good), it is proposed to adopt the product approach. The product approach begins by defining the main objective, generating a space of needs, taking into account the user and the context, and finally an organization of the needs is carried out [51]. For the project of an ROV system, and based on the product approach, the following activities are proposed for the “market” stage:

- Definition of the product and the market: define the objective of the generic use of the ROV, classifying it into research tasks or subsea operation tasks. The scope of the research or the sector of application must be specified. For this first stage, it is proposed to incorporate the taxonomy of problems of R.B. Frost [52], which establishes seven key factors for the organization and classification of problems from which the most suitable for this proposal are selected, being: (1) the type of entity being designed, (2) the degree of innovation involved, (3) the extent to which the designed entity can be conceptually decomposed into subsystems, (4) the availability of adaptable solution concepts, (5) the simplicity or complexity, (6) the degree of interaction within the solution and (7) the looseness or tightness of the constraints or requirements which the design must satisfy.
- Identification of reference users and collection of information: in this case, it is proposed to apply the requirements elicitation technique [53] to obtain the ideal technique for each ROV system project based on the information in the previous point. For the choice of an elicitation technique, there are attributes to consider, of which attention will be focused on the following attributes: level of available information, moment of application (in this case, early stage of the project), type of information to elicit (in this case needs), the degree of definition of the problem, the consensus between the users (experts) and the time constraint of the project. This methodology proposes 17 possible techniques, from which the most suitable ones will be selected according to the project. As a result of application, the application of open interviews and structured interviews with experts is recommended. Table 1 shows an example of basic questions to incorporate in a structured interview.
- Interpretation of user data from the need: the data obtained in the previous phase must be translated into user needs based on the UNE-EN 16271:2013 regulation [54]. Necessity is understood as those problems that users want to solve by using the ROV. It may also be of interest to collect information on user expectations, understood as the value that users expect to obtain from the product and the user experience, as well as a series of requirements. The needs analysis to define the PDS must be articulated and approved before proceeding with the following steps [55].
- Organization of needs and hierarchy: for the organization of needs, it is proposed to group them based on the information obtained, resulting in the following classification in the case of the ROV: basic, user, technical, innovative, corporate and regulatory framework needs. Subsequently, it is proposed to carry out a hierarchical analysis of needs in which they are defined based on the importance they have with respect to the objective of using the ROV. To carry out the hierarchy of needs, it is proposed to incorporate a binary dominance matrix, a matrix of paired comparisons, and in cases of greater complexity, the technique of the weighted technical value can be incorporated, understanding the complexity of the needs as the degree of difficulty in to satisfy that need. The weighting result of the needs has been included in Figure 9.

The subsequent definition that will be established based on the PDS implies asking the right questions. These questions must be answered by the internal or external customer in broad functional terms to allow the designer engineer the freedom to innovate [56].

In this stage, PDS are not proposed, but all the information necessary to establish the PDS is collected in the following specification stage.

#### 3.1.2. Design Core: The Product Design Specification

In this stage, it is proposed to incorporate the Methodology of Organizing Specifications in Engineering (MOOSE) of Morris and Stauffer [57]. This methodology starts from the requirements obtained from the users and the context as inputs to the engineering specifications which are translated into product specifications. For example, the need to have an ROV that is easy to load and unload can be translated into ROV size and weight specifications. MOOSE proposes organizing the specifications into four large areas: end user specifications and context (functionality, usability …), specifications of the legal regulatory framework (safety, environment, end of product life …), technical specifications (engineering practices, manufacturing processes, assembly and maintainability, available resources …) and corporate specifications (strategic management, finances, product portfolio, distribution systems, support …) [58]. Having the specifications classified based on MOOSE can streamline the process and facilitate the analysis of the requirements and their importance in the ROV design and development project.

For the next stage of Total Design, concept design, the functional requirements that belong to the area of MOOSE end-user requirements will have special relevance. Focusing on the functional specifications, it is proposed to incorporate the guidelines of the UNE-EN 16271:2013 standard [54] for ROV Functional Specification Document development. The application of this approach makes it possible to introduce and facilitate a competitive dialogue between the owners or managers of the project and the suppliers, since it proposes a well-founded approach and requires a tailored response. On the other hand, it allows the parties to appreciate the differences between the solutions proposed in the concept design stage to solve the needs and PDS, thus facilitating comparisons between various design alternatives.

Attention will be focused on the functional specifications (functional domain), since these are the ones that a priori are closely related to the sensory proposal (physical domain) of the ROV system. The functional specifications must be expressed as “verb + complement” with an attribute (unit of measurement), level (value) and tolerance (admissible variation) whenever the information is available. This same specification definition proposal can be extrapolated to all proposed ROV system specifications. It is proposed to categorize the functional requirements based on their origin or utility, thus obtaining utilitarian, operational, technical functions (manufacturing requirements) and those derived from compliance with regulations, as well as other possible ones related to the brand, recyclability. For the hierarchization of functions, the incorporation of the techniques applied in the previous point for the hierarchization of needs is proposed. Finally, it can be very useful to apply functional analysis and specification techniques such as the functional systems analysis technique (FAST) and object-oriented analysis (OOA).

To verify that the PDS correspond to the objectives, needs and expectations obtained in the “Market” stage, it is proposed to carry out a systematic analysis of the user information to improve the quality of the ROV concept to be developed. In a first phase, the relationships of the needs obtained with the PDS will be analyzed by creating a matrix in which the rows represent the needs and the columns the PDS (Figure 9). In addition to PDS proposed by Pugh [34], the generic PDS checklist proposed by Robin Kent is taken into account [56]. To weight the relationships between needs and PDS, a three-point scale is proposed, with a value of 1 being a “low relationship”, 3 being a “medium relationship” and 9 being a “high relationship”. In the proposed matrix, a column will be included where the relative importance of the needs is quantified based on the hierarchical analysis of needs previously proposed. This need importance value is useful for weighing the level of relationship between needs and PDS. This proposed matrix allows for quantifying the importance of the PDS to obtain a priority and hierarchy of PDS based on the needs in order to analyze competitive and innovation opportunities, plan the ROV to respond to the needs and opportunities and establish critical objectives values of the PDS.

Once the way to proceed is defined, an analysis of the PDS is carried out in detail, since each PDS must be characterized (Table 2). Within this analysis, attention is focused on the most important PDS (Figure 9) that may be related to the physical domain of the ROV sensors.

#### 3.1.3. Concept Design

Once the relevant PDS specifications have been defined, the next stage of the Total Design model is the conceptual design stage. The objective of this stage is to begin to define the physical domain of the ROV. In this case, the physical domain, also known as the domain of sub-systems and components, is limited to the sensor sub-system, which will consist of all the sensors to be included in the ROV. The sensors to be analyzed are those that are most closely related to the requirements of the MOOSE classification, focusing on the user context and end user requirements (usability, functionality and environment) and in the technical requirements (Figure 9). The sensor proposal is obtained from the previously defined bibliographical analysis, being the most representative in environmental and oceanographic research projects (Table 3).

Once the sensors of greatest interest in research tasks have been defined (Table 3) to satisfy the PDS obtained in Figure 9, the conceptual design stage will continue in the next point with the aim of modularizing the sensor sub-system.

### 3.2. Proposal of Sensorization through Total Design

Continuing with the conceptual design stage, it is proposed to carry out a modularization of the sensor system based on the PDS. The sensor system modulation strategy can facilitate the reconfiguration of the ROV system to suit different activities or tasks, as well as facilitate maintenance and repair tasks by adding, replacing or removing specific modules or components. This proposal is based on Simpson’s approach [59] for the development of system platforms and products based on modules (module-based). To achieve this modular proposal, similarity metrics are introduced that help establish the benefits of a family of products based on modules.

For the modularization, it is proposed to incorporate the Design Structure Matrix (DSM) technique, which is focused on the management of complexity in elements that are part of a system or product. The development of the DSM is based on establishing the elements that are part of a system or product and establishing possible relationships between them. There are several types of DSM, of which the DSM based on components (products) is of interest in this study, since it aims to establish relationships between components and functions in order to provide architectures and layout of systems and products [60]. Based on Pimmler and Eppinger [61], the main relationships to be analyzed in a DSM based on components can be of a spatial origin (need for closeness or orientation between two elements), material origin (need to exchange matter between two elements), informational origin (need to exchange information between 2 elements) and energy origin (need to transfer or exchange energy between two elements). In the case of DSM application for ROV sensorization, the relationships are established based on the function of the sensor and the type of information to be collected from the environment and from the ROV itself.

Regarding the sensors established in Table 3, the leak sensor and the internal pressure sensor must be located in the watertight and sealed section of the ROV into which water must not penetrate. In this area are the main electronic components such as the motor controller motherboard, batteries, etc. These sensors have not been introduced in the DSM since, due to specifications, they must be separated from the rest of the sensors. The other sensors to be analyzed in the DMS can be located in the wet areas of the ROV and areas in direct contact with the marine environment.

**Figure 10:**
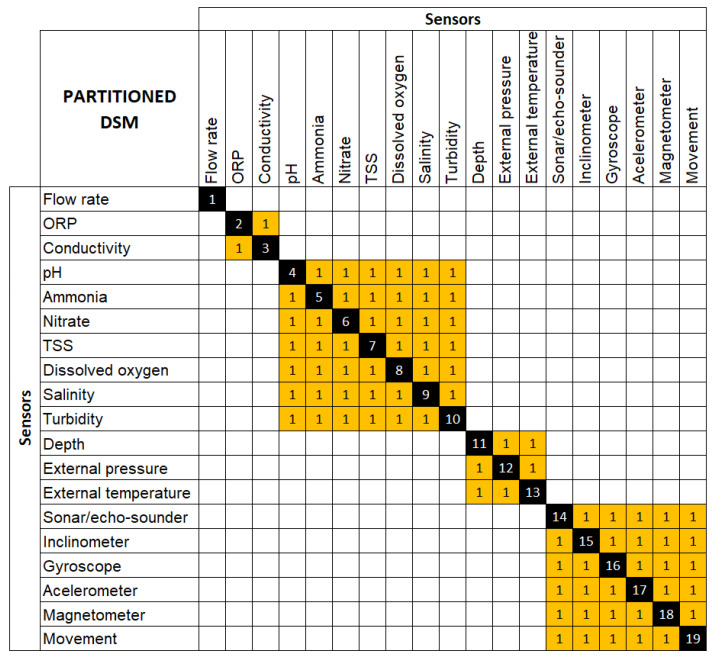
DSM application result.

After establishing the matrix of sensor relationships and applying the DSM clustering algorithm, the matrix in Figure 10 is obtained. The objective of the clustering algorithm is to maximize the interactions within the cluster (module) and minimize the interaction between clusters (modules). The basic operation of the algorithm is to reorder the rows and columns of the interaction matrix so that all scores (ratios) are as close as possible to diagonal or form a tight cluster with other scores.

After performing the clustering of the DSM, four modules and an independent sensor are obtained. A module is understood as a building block of a larger system or product that has a specific function and well-defined interrelationships [62].

- Flow module: one sensor (Sensor 1). This sensor is independent from the rest since for the measurement of speed of water, it is common for sediments to be transported, which would require other specific components that the rest of the sensors do not require. If sediment transport is not necessary, it could be included in the water analysis module.
- Solubility module (ORP oxidation and conductivity): two sensors (sensor 2 and 3 in Figure 10). This module is specific for the analysis of oxidation and conductivity, which usually correspond to a detailed analysis of water that is not always required.
- Water analysis module: seven sensors (sensor 4 to 10 in Figure 10). This module encompasses the analysis of the basic parameters of water that can be the object of research. Within this module there may be sub-modules such as the sub-module formed by the TTS sensor and the turbidity sensor since the TTS sensor provides a complementary measure of turbidity.
- Depth/pressure module: three sensors (sensor 11 to 13 in Figure 10). This module is intended to obtain basic information on the environment, such as the pressure of the ROV (which is a function of depth) and the temperature of the water.
- Movement control module: six sensors (sensor 14 to 19 of Figure 10). This module is intended to obtain information on the displacement of the ROV in the environment such as the position, speed, inclination of the vehicle.

Once the modularity of the sensor group is obtained, the work approach can be extrapolated to the rest of the physical elements or components in such a way that they provide a design briefing to define the layout of the ROV and its physical architecture.

This research defines and provides methodological tools for the early stages of the design and development of ROVs, since based on the bibliographic study carried out, there has been an increase in studies related to ROVs that are very focused on the detailed design stage, definition of CDS and simulation, with there being a lack of approaches to defining PDS and in their conceptualization. As a result of the applications, being a complex product, attention has been focused on the sensors, since a large proportion of previous studies focused on the detailed design and manufacturing stage of said elements. The proposal for market study, the definition of PDS and conceptualization can be extrapolated to the propulsion system, casings and structural elements, control system, etc. The idea is that, after defining these early stages, they continue with the stages proposed in the Pugh model, such as the stage of detail design, manufacture and sell, of which there is more developed information in the bibliography.

The possibility of obtaining a modular ROV can help to design a basic ROV which, through the coupling or uncoupling of modules, can be adapted to different fields of research and to different areas of industrial work. This modular conceptualization of ROVs could help to introduce this product within the “domestic” product market, since today there is no ROV proposal focused on a non-expert user whose main needs may be to have fun and obtain basic information about the environment. Products with a similar development, such as drones, have a more diversified offer and serve the non-expert user, regardless of the uses that drones have in research and in industrial applications.

This type of approach and proposal can bring the development of ROVs closer to the general public and encourage the interest of a non-expert user in the marine environment of seas and oceans and in more accessible environments such as rivers, lakes and lagoons.

The bibliographic analysis carried out concludes that there is a research gap in the development of ROVs from the discipline of industrial design engineering. There is a research opportunity that improves the organization and application of stages such as market research, definition of specifications and conceptual design in ROV design and development projects.

The design and development process in engineering requires a comprehensive approach. The use of the Total Design model is proposed, which synthesizes the main activities together with recurring aspects in engineering projects in the form of product design specifications.

In this work, attention is focused on the stages of “market”, “specification” and “concept design”.

To address the “market” stage, we propose carrying out an analysis focused on the needs of users regarding a ROV system. For the ROV system, being a highly technological product and not being a product of great consumption or domestic consumption, we propose adopting the product approach. For the project of an ROV system, and based on the product approach, the following activities are proposed for the “market” stage: definition of the product and the market, identification of reference users and information gathering, interpretation of the data of the user from the need, and organization of the needs and hierarchy.

In the “specifications” stage, we propose incorporating the Methodology of Organizing Specifications in Engineering. Thus, this is based on the requirements obtained from the users and the context as input to the engineering specifications, which are translated into product specifications. Focusing on the functional specifications, we propose incorporating the guidelines of the UNE-EN 16271:2013 standard for the development of the functional specifications of the ROV system. To verify that the PDS correspond to the objectives, needs and expectations obtained in the “market” phase, it is proposed that we carry out a systematic analysis of user information to improve the quality of the ROV concept to be developed.

Once the specifications of the relevant PDS have been defined, the conceptual phase of the design core is addressed. The proposal of sensors to analyze includes the most representative in environmental and oceanographic research projects. It is proposed that we carry out a modularization of the sensor system based on the PDS. This modularization proposal has been carried out using the DSM technique, obtaining four modules and an independent sensor.

## 4. Conclusions

The limitations to which this work has been subject are expressed below and suggestions for future work are made.

This research is based on a bibliographical study, with the limitations that this entails. These limitations derive from the type of sources consulted, their selection criteria and the limits to the work. There may be works that are not directly from the authors, but manuals that compile several works or authors. Similarly, it is advisable to read the works in their original language when possible. Regarding the selection criteria of the works, it is important that these are not the result of chance or bias of the researcher. Finally, the limitations of the review include a time interval, excluding the rest. It is also necessary to mention as a limitation the relevance and quality of the sources, tools and search strategies used.

On the other hand, the entire proposal of this paper is focused on the initial stages of the design and development process of new products. In this work, attention has been focused on the sensors, this being a limitation or initial boundary condition of our research.

We can state as future works extrapolating the market study proposal, definition of PDS and conceptualization to other systems, such as the propulsion system, frame and structural elements and control system, among others.

Another possible future work is that after the definition of the early stages, the stages proposed in the Pugh model are continued, such as the phase of detail design, manufacture and sell.

A very interesting and tangible future work is the design of a basic ROV, which by coupling or uncoupling modules can be adapted to different fields of research and to different areas of industrial work.

The proposed modular conceptualization of ROVs could also be useful in the design of “domestic” products, focused on a non-expert user whose main needs may be to have fun and obtain basic information about the environment. This design of recreational vehicle, for non-professional underwater photography or exploration, for instance, can bring the development of ROVs closer to the general public and encourage the interest of a non-expert user in the marine environment of seas and oceans and in more accessible environments such as rivers, lakes and lagoons.

## Figures and Tables

**Table 1:** Example of an extract of questions to elicit needs in relation to the PDS of the next stage.

Questions for Needs Elicitation
Will the vehicle operate in fresh or salt water?
Will be the ROV in controlled environments or in natural spaces?
What species can be found in the environment?
Should regular or planned maintenance be performed?
What type of maintenance will be performed?
What components present increased exposure?
What is the intended use of the product?
What information is collected with the ROV?
What is the previous experience using ROVs?
Are there speed restrictions?
What is the maximum inspection time?
Will special materials be necessary?
Is there a risk of collision?
Are there restrictions on the application of materials according to the environment?

**Table 2:** Proposal for the characterization of the obtained PDS.

Need	PDS	Specification	Units
Obtain information of water	Environment	Temperature	°C
Obtain information of sea bed		Pressure/depth	bar/m
Obtain information of submerged structures		Water analysis	multiple units
Obtain information of marine biodiversity		Distance measurement	m
Allow displacement in the environment		Detection of objects and fauna	·
Knowledge of state	Maintenance	Temperature	°C
Be easy to disassemble		Pressure	bar
Be resistant to environment conditions		Internal state	·
Use suitable materials for water		Service life	years
Allow displacement in the environment	Performance	Speed	m/s
Have access to energy source		Depth	m
Knowledge of internal state		Power	W
Knowledge of position		Positioning (USBL)	m
Communicate information		Autonomy	h
Avoid turbulence in the environment	Materials	Impact resistance	kJ/m²
Be easy to manufacture		Weight	kg
Use suitable materials for water		Pressure	bar
Be resistant to environment conditions		Temperature	°C
Be resistant to fauna		Cost	€
Communicate information	Users	Usability	·
Be easy to assemble		Experience	·
Be easy to operate		Control	·
Be easy to transport		Operation	·

**Table 3:** Proposal of sensors for the obtained PDS.

MOOSE Clasification	Sensor	Function	Units
User Context and End user. Usability. Functionality. Environment.	Flow rate	Measurement of speed of water by studying flow patterns.	m/s
	Oxidation-Reduction Potential (ORP)	Measurement of the ability of a solution to act as an oxidizing or reducing agent.	mV
	pH	Measurement of concentration of hydrogen ions in water, a measure of the acidity or alkalinity of water.	pH
	Ammonia	Measurement of concentration of ammonia in water.	mg/L
	Nitrate	Measurement of concentration of nitrate in water.	mg/L
	Turbidity	Measurement of turbidity of water and monitoring the formation of precipitates and populations of algae.	NTU
	Total Suspended Solid (TTS)	Measurement of total concentration of suspended solids in water, a complementary measure to turbidity.	g/L
	Dissolved oxygen	Measurement of concentration of dissolved oxygen in water.	mg/L
	Conductivity	Measurement of the ability of a solution to conduct electricity.	S/m
	Salinity	Measurement of the content of dissolved salts in water.	mg/L-ppm
Technical	Depth	Depth range control.	m
	External pressure	Pressure measurement according to the depth reached.	bar
	External temperature	Measurement of water temperature.	°C
	Sonar/echo-sounder	Obstacle and fauna detection.	acoustic pulse
	Inclinometer	Measurement of the inclination and roll in the vehicle.	°
	Gyroscope	Measurement of angular velocity of the vehicle in one or more axes.	°
	Acelerometer	Measurement of vehicle acceleration in one or more axes.	m/s^2^
	Magnetometer	Measurement of the strength or direction of the magnetic field.	T (tesla)
	Movement	Provide information about the stability and heading of the moving vehicle.	·
	Leak sensor	Allows the detection of leaks in the electronic POD.	·
	Internal pressure	Measurement of pressure inside the POD.	bar

## Data Availability

No applicable.
